# Segmentation of the millipede trunk as suggested by a homeotic mutant with six extra pairs of gonopods

**DOI:** 10.1186/1742-9994-11-6

**Published:** 2014-01-17

**Authors:** Nesrine Akkari, Henrik Enghoff, Alessandro Minelli

**Affiliations:** 1 Natural History Museum of Denmark (Zoological Museum), University of Copenhagen, Universitetsparken 15, København Ø DK-2100, Denmark; 2 Department of Biology, University of Padova, Via Ugo Bassi 58 B, Padova I-35131, Italy

**Keywords:** Segmentation models, Ectopic gonopods, Transcription factor, Positional marker, Segmentation genes

## Abstract

**Background:**

The mismatch between dorsal and ventral trunk features along the millipede trunk was long a subject of controversy, largely resting on alternative interpretations of segmentation. Most models of arthropod segmentation presuppose a strict sequential antero-posterior specification of trunk segments, whereas alternative models involve the early delineation of a limited number of ‘primary segments’ followed by their sequential stereotypic subdivision into 2^n^ definitive segments. The ‘primary segments’ should be intended as units identified by molecular markers, rather than as overt morphological entities. Two predictions were suggested to test the plausibility of multiple-duplication models of segmentation: first, a specific pattern of *evolvability* of segment number in those arthropod clades in which segment number is not fixed (e.g., epimorphic centipedes and millipedes); second, the occurrence of *discrete multisegmental patterns due to early, initially contiguous positional markers*.

**Results:**

We describe a unique case of a homeotic millipede with 6 extra pairs of ectopic gonopods replacing walking legs on rings 8 (leg-pairs 10-11), 15 (leg-pairs 24-25) and 16 (leg-pairs 26-27); we discuss the segmental distribution of these appendages in the framework of alternative models of segmentation and present an interpretation of the origin of the distribution of the additional gonopods.

The anterior set of contiguous gonopods (those normally occurring on ring 7 plus the first set of ectopic ones on ring 8) is reiterated by the posterior set (on rings 15-16) after exactly 16 leg positions along the AP body axis. This suggests that a body section including 16 leg pairs could be a module deriving from 4 cycles of regular binary splitting of an embryonic ‘primary segment’.

**Conclusions:**

A very likely early determination of the sites of the future metamorphosis of walking legs into gonopods and a segmentation process according to the multiplicative model may provide a detailed explanation for the distribution of the extra gonopods in the homeotic specimen. The hypothesized steps of segmentation are similar in both a normal and the studied homeotic specimen. The difference between them would consist in the size of the embryonic trunk region endowed with a positional marker whose presence will later determine the replacement of walking legs by gonopods.

## Introduction

**Figure 1:**
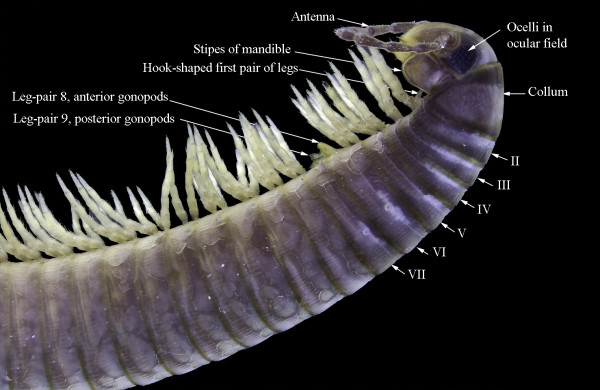
***Ommatoiulus moreleti*, normal male: head and anterior body rings; lateral view.**

The characteristic mismatch between dorsal and ventral trunk features along the trunk of millipedes (Diplopoda) defies simple interpretations in terms of segments as archetypical building blocks of the arthropod body and also challenges traditional views on segmentation. The typical antero-posterior sequence of postcephalic units includes a legless collum, three rings with one pair of walking legs each, and a more or less numerous series of rings with two pairs of legs each. In the typical, cylindrical millipedes which constitute the large clade Juliformia, the anterior (8th) or both (8th and 9th) pairs of legs on trunk ring 7 are replaced in the male by gonopods, strongly modified sexual appendages (Figure 1).

A naïve question that in the past has been repeatedly floated is, whether a ring with two pairs of legs is to be interpreted as a single segment, or as a double segment. The second alternative has been generally favoured, as it is more easily brought into agreement with the basic assumption that the trunk of arthropods is fundamentally composed of segments with one pair of appendages each. As a consequence, the articulated rings of the millipede trunk are frequently called diplosegments, or diplosomites.

However, current understanding of arthropod segmentation suggests that framing the question in terms of equivalence of millipede rings to conventional (or ‘archetypical’) arthropod segments is not necessarily meaningful. On the one hand, studies on the patterns of expression of segmentation genes in the pill millipede *Glomeris marginata*[1] have shown that the processes of segmentation in the dorsal and the ventral halves of the body are decoupled on the level of the expression of segment polarity genes. As a consequence, trunk rings do not emerge as primary units of trunk organization, but as secondary products of morpho-functional coupling of primarily independent serial structures [2]. Up to now, no corresponding studies have been performed on representatives of the clade Juliformia, but these are likely to agree in this mechanism of segmentation with the Pentazonia, to which *Glomeris marginata* belongs. It is not known how far independent dorsal vs. ventral segmentation occurs throughout the Arthropoda; besides *Glomeris*, anyway, it has been recorded in a crustacean, the tadpole shrimp *Triops longicaudatus*[3], and in the spider *Cupiennius salei*[4]. On the other hand, among the many genes involved in the segmentation of arthropods, some genes have been discovered to be expressed, at least transiently, in bisegmental, rather than segmental patterns. This peculiar pattern of expression has been observed in many insects, including *Drosophila*[5], the grasshopper *Schistocerca gregaria*[6] and the lesser flour beetle *Tribolium castaneum*[7], but also in the two-spotted spider mite *Tetranychus urticae*[8], in the geophilomorph centipede *Strigamia maritima*[9,10] as well as – significantly – in the pill millipede *Glomeris marginata*[11].

It is still a matter of dispute whether these transient bisegmental patterns belong to the primitive mechanism of arthropod segmentation, or have evolved multiple times within the phylum, perhaps involving different cascades of control of gene expression. Most authors, e.g. [12-14] are cautious in according either generality or key mechanistic importance to these patterns, but diverging views have been also expressed [14]. According to a model first formulated by Maynard Smith [15], more extensively articulated by Minelli and Bortoletto [16] and subsequently revised by Minelli [17], in arthropods whose trunk is formed by a high number of segments, such as centipedes and millipedes, segmentation would involve more than one cycle of duplication, starting with a small, and probably quite constant number of primary segmental units. A multiplicative model of segmentation does not necessarily imply an overt morphological subdivision of tissue, a process of which there is no evidence in any arthropod studied to date. However, molecular markers corresponding to N, 2 N, 4 N, 8 N, 16 N units might well be expressed before segments are visible as morphological entities. This is indeed implied by our interpretation of the segmental distribution of gonopods in the *Ommatoiulus* homeotic specimen described in this paper.

Up to now, developmental genetics has not offered positive evidence in favour of duplications extending beyond a first cycle. However, circumstantial evidence offered by comparative morphology is more convincingly explained by a multiple duplication model than by any alternative models and suggests the need for further investigations.

There are two predictions, at least, in respect to which the multiple duplication model performs much better than alternative models of arthropod segmentation.

A first kind of prediction is about the *evolvability* of segment number in those arthropod clades in which, as in epimorphic centipedes and in millipedes, segment number is not fixed. If, in the course of embryonic development, the hypothesized primary segments undergo a few cycles of splitting, thus giving rise to a rapidly growing number of units (as a first approximation, N, 2 N, 4 N, 8 N…), it might not be that difficult for this mechanism to be occasionally repeated one extra time, thus producing individuals with a roughly duplicated number of segments, without any intermediate between the original number and the duplicated one. Among centipedes, *Scolopendropsis duplicata* Chagas, Edgecombe and Minelli, 2008, very similar to *Sc. bahiensis* (Brandt, 1841) except for the approximately double number of segments fits well into this model of ‘saltational’ morphological evolution [18].

A second kind of prediction is about *discrete multisegmental patterns due to early positional markers*. Periodic, multisegmental patterns may actually depend on a diversity of causes. As illustrated by Enghoff [19] on trans-segmental colour patterns in millipedes, these patterns often correlate with the successive batches of segments added with subsequent moults. This is a reasonable expectation, as the colour pattern is likely determined concurrently with the full development of subsequent batches of segments. However, the segmental distribution of multisegmental patterns does not always agree with the pattern of stepwise addition of segments at the posterior end of the trunk; if these patterns are due to an early expression of positional markers, their segmental distribution may allow formulating suggestions about segmentation mechanisms.

In millipedes, gonopods appear late in post-embryonic development. Indeed, these specialized appendages are the product of a kind of ‘non-systemic metamorphosis’ [20,21] of appendages first expressed as normal walking legs during previous post-embryonic stadia. However, their position along the trunk is very likely fixed much earlier, during embryonic development, when the segmental organization of the trunk is only partially deployed. Therefore, a millipede with ectopic, extra pairs of gonopods may offer precise suggestions as to the process of segmentation.

In this paper we describe a field-collected male of the julid millipede *Ommatoiulus moreleti* (Lucas, 1860) (Julida: Julidae) with as many as six extra pairs of gonopods and discuss the segmental distribution of these appendages in the framework of alternative models of segmentation. The multiple duplication model offers a detailed, although tentative explanation for this unique phenotype, whereas no prediction about the segmental distribution of the extra gonopods can be derived from alternative models.

## Results

### General description of the homeotic specimen

**Figure 2:**
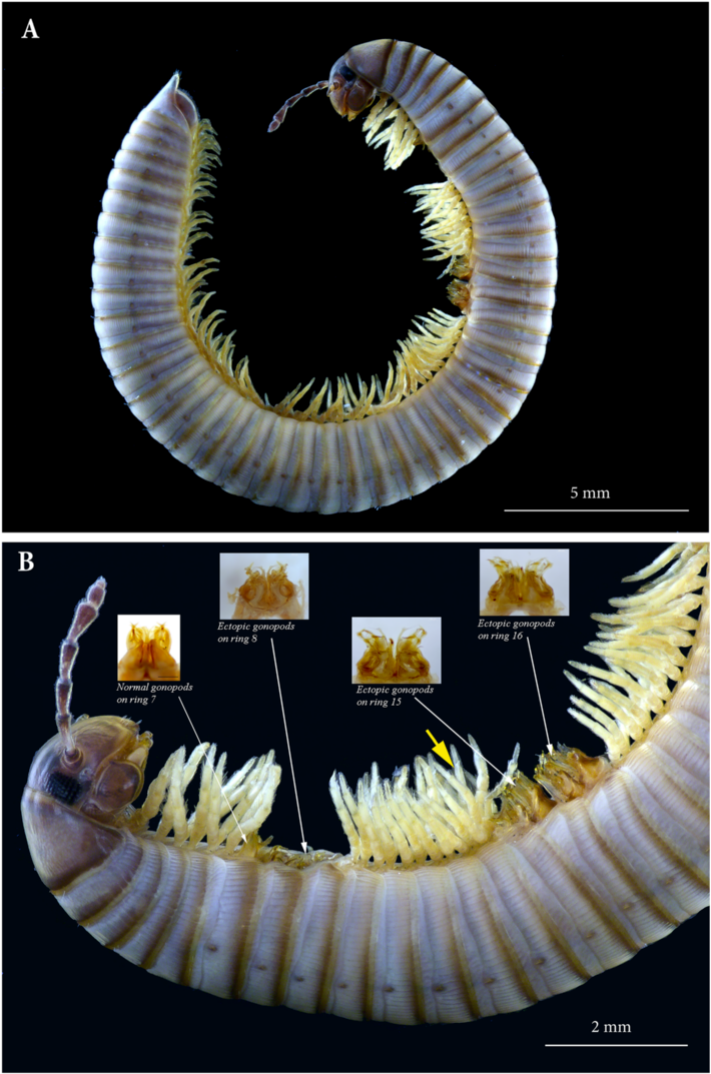
***Ommatoiulus moreleti*, homeotic male with extra pairs of gonopods. (A)** The specimen in toto, lateral view. **(B)** Close-up of the anterior half (head and first 17 body rings). Yellow arrow points to a set of four leg pairs corresponding to a single body ring (described in this article as ring 13–14).

The studied homeotic specimen is a mature male of *Ommatoiulus moreleti* at post-embryonic stadium 10 (according to Enghoff et al. [22]) with 9 vertical rows of ocelli and 46 podous rings + telson (there are no apodous rings – an unusual condition in Julida [22]); maximum vertical diameter at mid-body 2.6 mm. The specimen presents the secondary sexual characters common for julids consisting of the presence of a well-developed mandibular stipital lobes and a hook-shaped first pair of legs (Figure 2).

Besides the six extra (ectopic) pairs of gonopods described below, the specimen presents further morphological anomalies. Its left antenna is atrophied, reduced to two antennomeres, of which the second is rudimentary and reduced in size to nearly half the normal one, apically tapering in a blunt rounded apex (Figure 2A).

Moreover, four pairs of normal walking legs (20th to 23rd pairs) are attached to one ring, rather than two, this single ring corresponding to rings 13 and 14 of a normal specimen. This circumstance, although spatially limited, adds to the evidence pointing to a degree of decoupling between dorsal and ventral segmentation in millipedes. As to the presence of a single ring where two distinct rings would be expected, to match a set of four pairs of appendages, two alternative hypotheses could be suggested in principle. Indeed, a double ring is possibly the product of a local extra fusion of embryonic dorsal segmental units [2] but, alternatively, it might be a later, morphological expression of a locally truncated pattern of segmentation, if the latter is laid down through a multiplicative process of distribution of segmental markers. Our suggestion in favour of the latter model of segmentation is based, however, on the segmental distribution of ectopic gonopods, as discussed below, whereas we do not have strong arguments for either tentative explanation of the presence of the double ring. In comparative terms based on the sequence of ventral appendages (walking legs and gonopods), this ring represents anyway the equivalent to rings 13 + 14 of a normal millipede.

The most conspicuous peculiarity of the specimen we are describing here is the presence of six extra pairs of posterior gonopods replacing the walking legs of rings 8 (leg-pairs 10 and 11), 15 (leg-pairs 24 and 25) and 16 (leg-pairs 26 and 27).

On each of the three body rings with two sets of abnormal posterior gonopods, generally the posterior set is a bit larger than the anterior; the latter more constricted and reduced.

There is an overall loss of structures from anterior to posterior, most visibly on the paracoxites reduced to slender processes on the posteriormost ectopic gonopods. Similarly, the solenomerites of the last pair of ectopic gonopods on ring 16 are highly reduced.

There is generally a loss of symmetry in the ectopic gonopods as evidenced by the presence of additional/rudimentary and not fully developed processes.

Except for the normal posterior gonopod on ring 7, where spermatozoa were detected (cf. [23]), all the foveae were completely empty.

It is quite remarkable that the extra gonopods all show the typical (although more or less modified) morphology of posterior gonopods whereas the anterior gonopods are not at all duplicated.

### Description of gonopods

**Figure 3:**
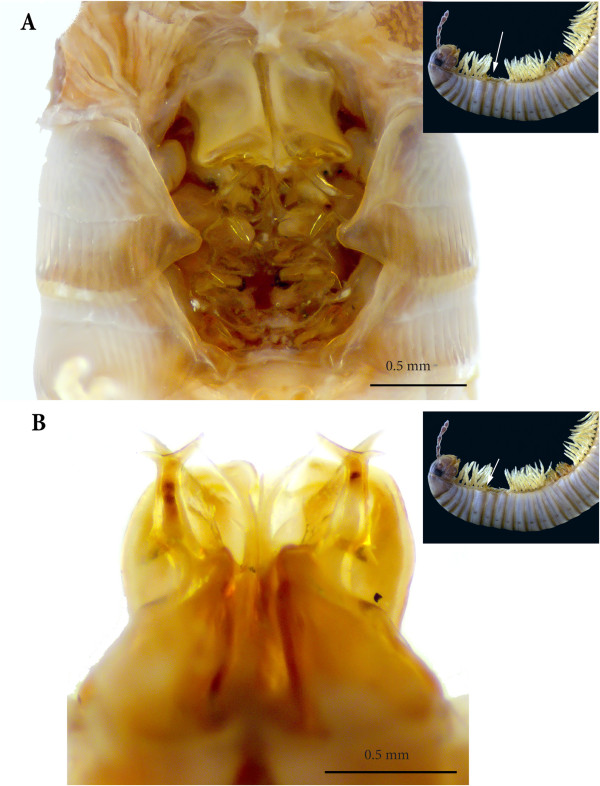
**Anterior sets of gonopods. (A)** Gonopods on rings 7–8, antero-apical view. **(B)** Gonopods on ring 7, posterior view.

**Figure 4:**
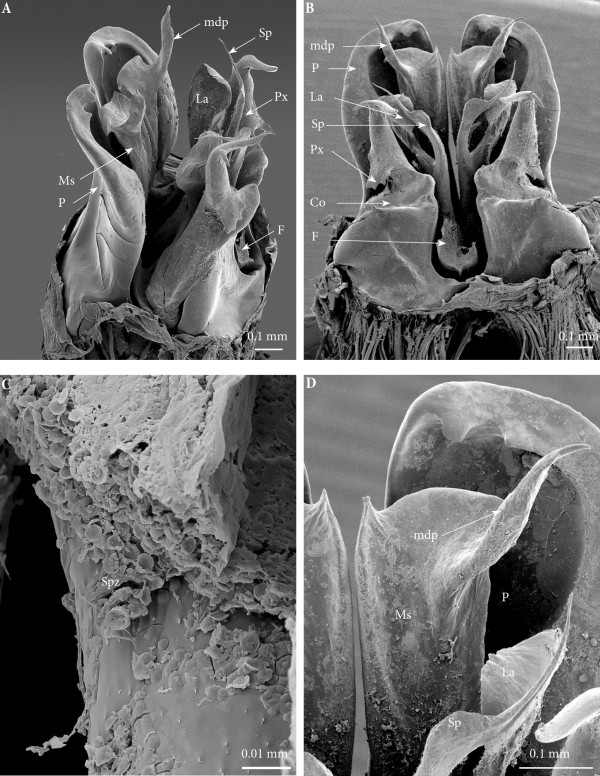
**Gonopods on ring 7, SEM. (A)** Latero-apical view. **(B)** Posterior view. **(C)** Spermatozoa in fovea. **(D)** Close-up of the distal part of the left gonopod, posterior view.

*Gonopods on ring 7* (Figures 3B and 4)*.* Normal, complete set showing all the structures usual for *O. moreleti*, i.e., anterior gonopod or promerite, and posterior gonopod. Promerite (**P**) broad, with parallel margins, apically rounded and on the posterior surface with two small apical tooth-like processes and a broad lateral ridge which delimits a posterior concavity. At the base of the lateral ridge a conspicuous ovoid telopodite remnant is inserted. Posterior gonopod: mesomerite (**Ms**) large, distally broadened and with a subapical slender process originating from the sublateral margin, distally tapering in a pointed apex pointing distolaterad (**mdp**). Apical margin of mesomerite rounded, with a mesal small acuminate process pointing distad and a curved lateral process pointing basad. Solenomerite broad and complex comprising a short slender process pointing meso-anteriad (**msp**), and a main posterior branch distally bifurcated into a lateral broad lamella (**La**) and a mesal slender process (**Sp**) distally bulging out and curving laterad in an aciculate distal part, pointing distolaterad. The base of the solenomerite lodges the fovea (**F**) from which departs the seminal groove which runs and apically opens at the tip of the process **Sp**. Spermatozoa (**Spz**) were seen in the fovea (Figure 4C). The paracoxite (**Px**) emerges from a reduced coxite (**Co**) and consists of an enlarged plate, apically reduced and bearing two apical slender processes and a broader basal one pointing laterad and showing several serrations.

**Figure 5:**
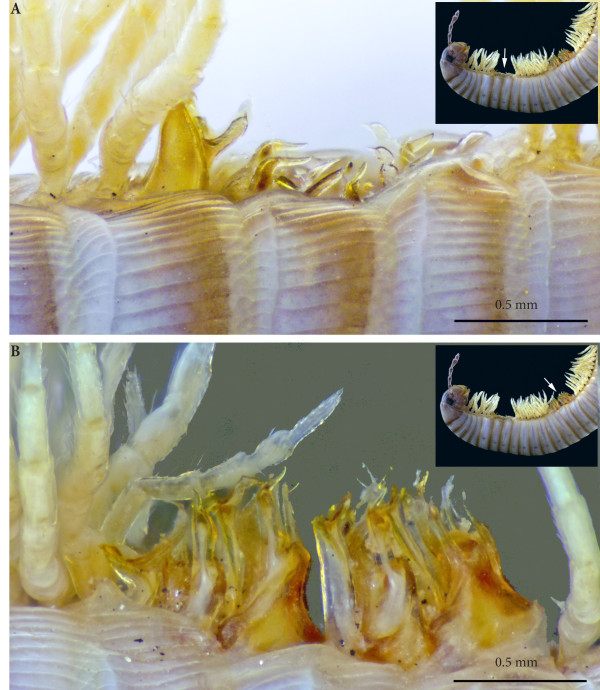
**Sets of gonopods, in situ, lateral view. (A)** Gonopods on rings 7–8. **(B)** Ectopic gonopods on rings 15–16.

**Figure 6:**
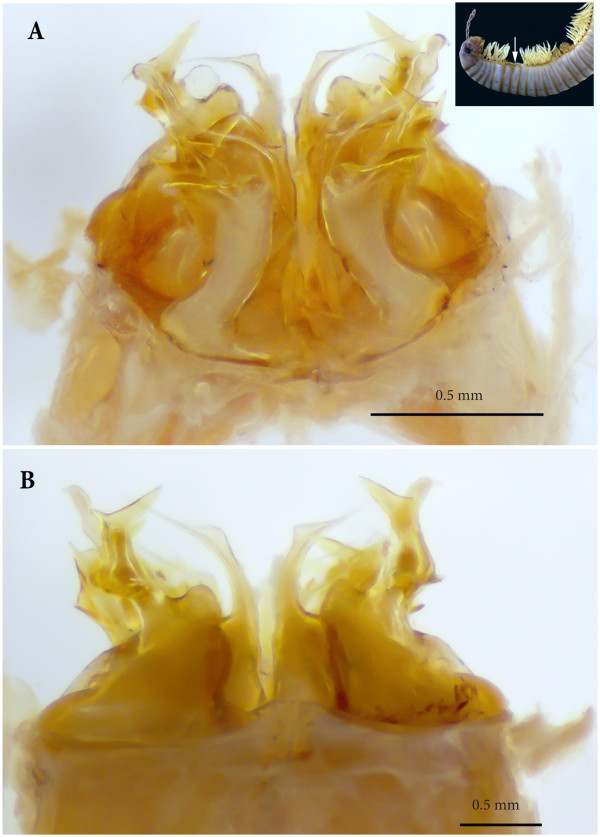
**Ectopic gonopods on ring 8, in toto. (A)** Antero-apical view. **(B)** Posterior view.

**Figure 7:**
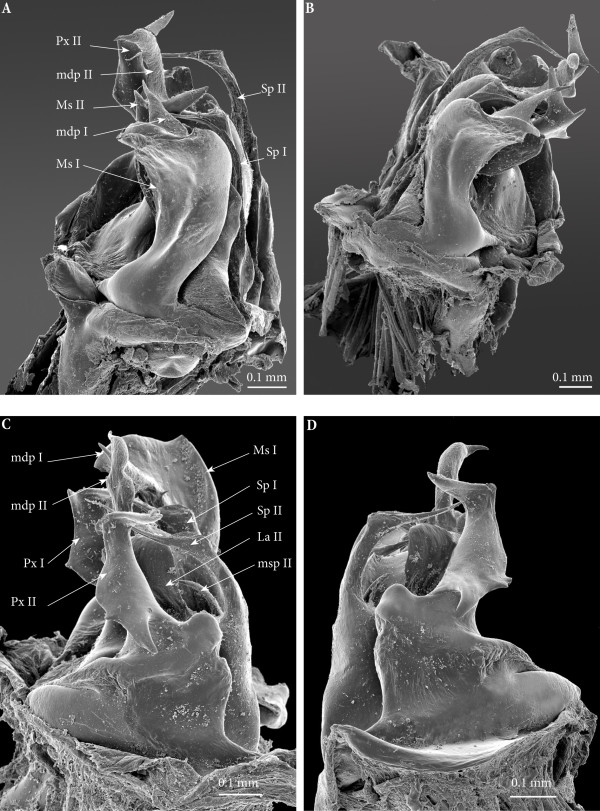
**Ectopic gonopods on ring 8, SEM. (A)** Left gonopod, antero-apical view. **(B)** Right gonopod, antero-apical view. **(C)** Left gonopod, postero-apical view. **(D)** Left gonopod, posterior view.

**Figure 8:**
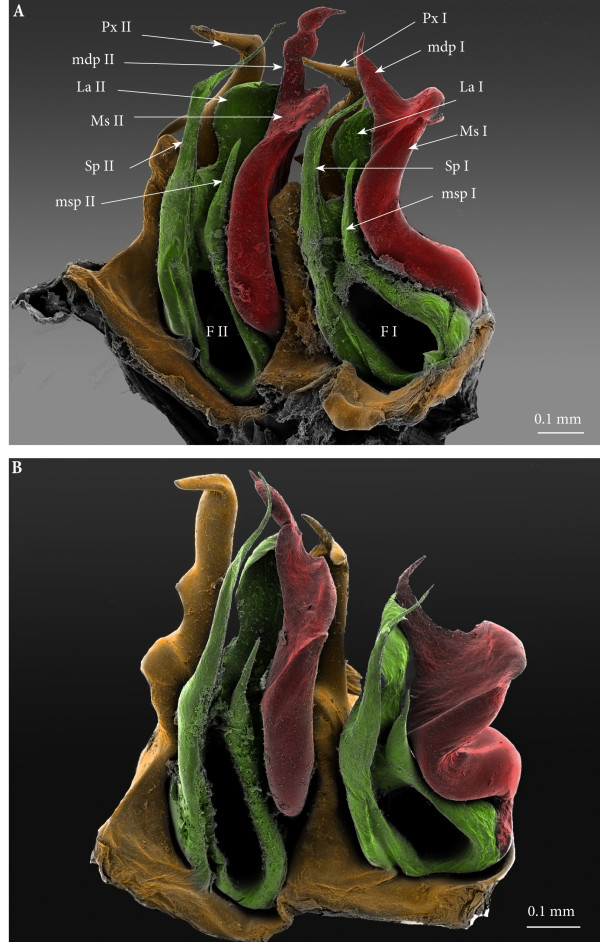
**Right ectopic gonopods on rings 8 and 15, mesal view, SEM, colours added. (A)** Right ecotopic gonopods on ring 8. **(B)** Right ectopic gonopods on ring 15. Red, mesomerites; Green, solenomerites; Golden yellow, paracoxites.

**Figure 9:**
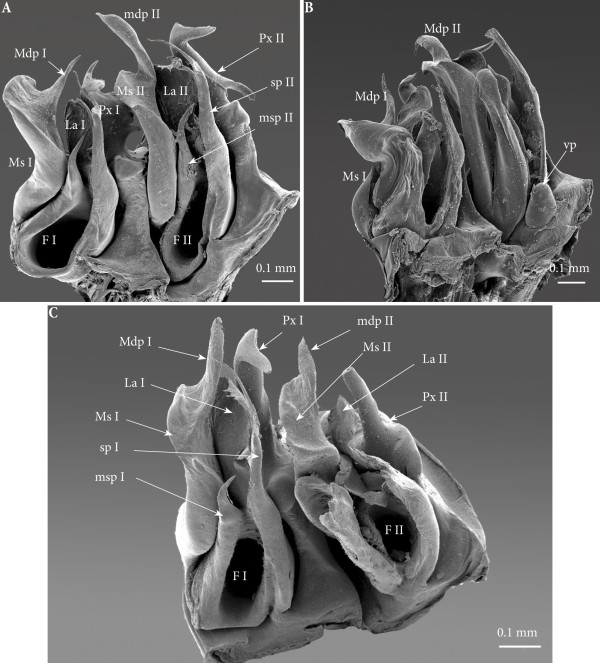
**Left ectopic gonopods on ring 8, 15, 16. (A)** Ectopic gonopod on ring 8, meso-apical view. **(B)** Ectopic gonopod on ring 15, mesal view. **(C)** Ectopic gonopod on ring 16, meso-apical view.

**Figure 10:**
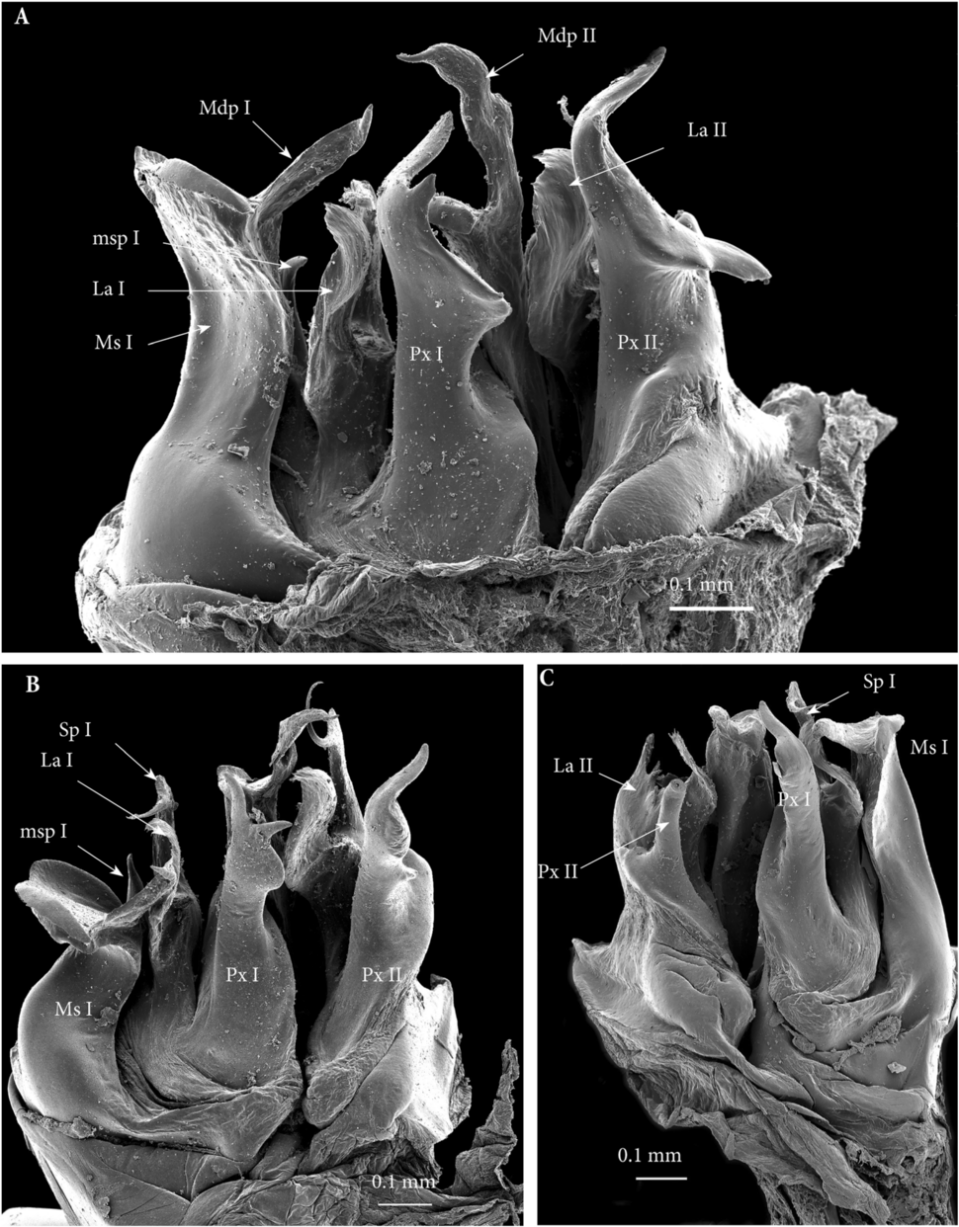
**Latero-apical view of ectopic gonopods on ring 8, 15, 16. (A)** Right ectopic gonopod on ring 8. **(B)** Right ectopic gonopod on ring 15. **(C)** Left ectopic gonopod on leg 16.

*Ectopic gonopods on ring 8* (Figures 3A; 5A; 6; 7; 8A; 9A and 10A)*.* Body ring 8 carries two consecutive and closely connected pairs of distorted posterior gonopods, presenting normal anterior-posterior polarity and consisting (from anterior to posterior) of

• Mesomerites I broadened and compressed anterobasad, distally bent anteriad; a process (**mdp)** with a broader base connecting to mesomerite especially on the right gonopod, shorter than normal, apically acuminate and pointing posteriad instead of laterad on both (Figures 6A; 7A and B).

• Solenomerites I composed of the three main processes described above but much reduced in size and with a completely empty fovea (Figures 8 and 9A).

• Coxites I compressed, distally more rounded, mesally with a prominent rounded distal margin. Paracoxites I showing most of the processes described above. However, apically the lateral process is much smaller, reduced to a small tip; and basally bearing a small lateral tooth and a protruding pointed process which is slenderer and not serrated (Figures 7C, D; 8A; 9A and 10A).

• Mesomerites II more or less of the same size as the preceding ones, bearing a much larger (compared to mesomerites I) process (**mdp)**, pointing anteriad (Figures 8A and 9A). On the right mesomerite, **mdp** is bi-articulated, with a constriction in the middle separating two subequal processes, the apical-most narrowed in a claw-like tip (Figure 8A).

• Solenomerites II complete, with significantly larger processes (compared to solenomerites I), showing a broader lamella (**la**), a larger (**msp)** and a more developed and longer process (**Sp)** (Figures 8A and 9A). Foveae empty.

• Coxites II distorted, shorter than normal, with a prominent round mesal apical margin. Paracoxites II slightly swollen laterally, apically strongly narrowed and bearing the two processes (the lateral one broken), basally with a small lateral tooth and a protruding process devoid of serrations (Figure 7C and D).

**Figure 11:**
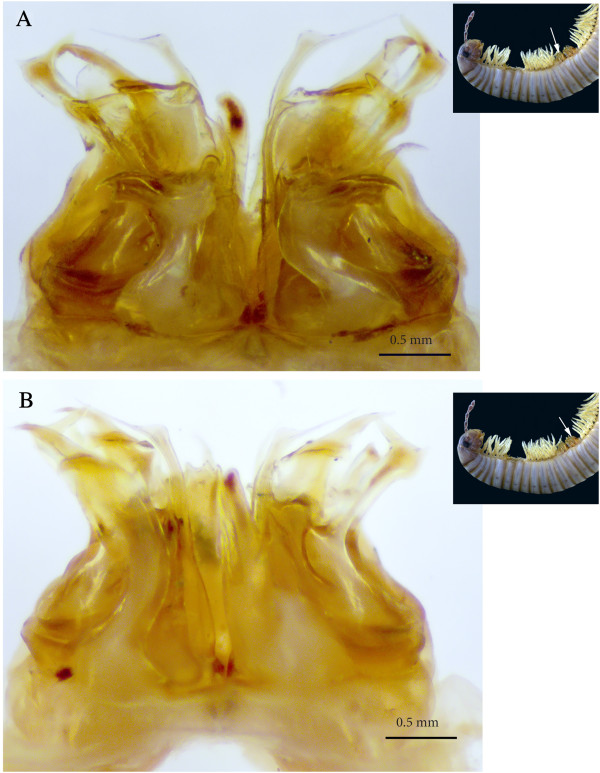
**Ectopic gonopods on rings 15 and 16, in toto. (A)** Antero-apical view. **(B)** Posterior view.

**Figure 12:**
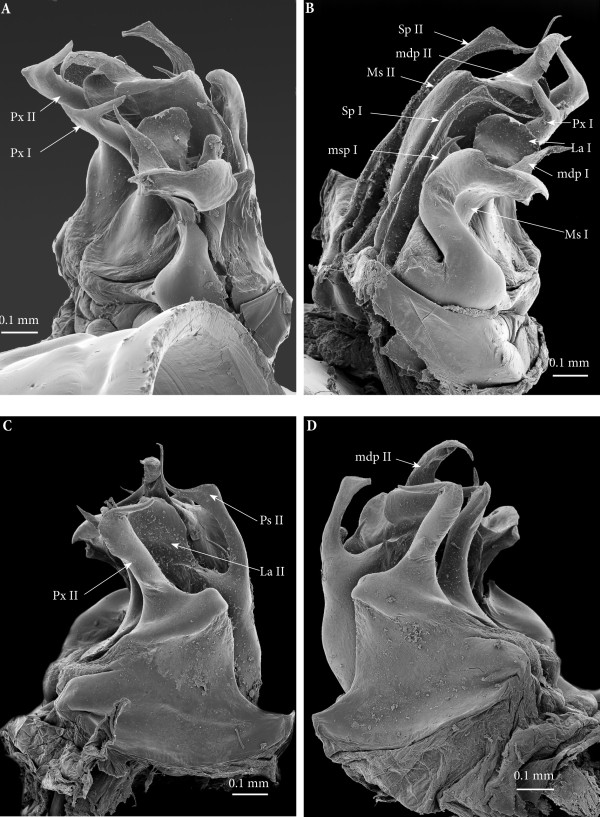
**Ectopic gonopod on ring 15, SEM. (A)** Left gonopod, antero-apical view. **(B)** Right gonopod, anteromeso-apical view. **(C)** Left gonopod, posterior view. **(D)** Left gonopod, posterior view.

*Ectopic gonopods on ring 15* (Figures 5B; 8B; 9B; 10B; 11A and 12). Two consecutive connected distorted posterior gonopods placed on ring 15, presenting normal anterior-posterior polarity, but asymmetrical and consisting (from anterior to posterior) of

• Mesomerites I: right mesomerite short, half as long as normal and very sinuous (Figures 12A, B; 8B and 9B) with a broad base, narrowing at mid-length and distally enlarged mesolaterad; process (**mdp**) with a broad base, distally tapering, curved and pointing anteriad. Left mesomerite showing modifications on the mesal side which consists in the presence of an additional mesal process (Figures 9B and 12A).

• Solenomerites I reduced, bearing processes **msp**, **Sp**, **La**, all reduced in size. Right solenomerite complete (Figure 8B) while the left is lacking the antero-mesal process (**msp)** which is absent or not fully formed (Figure 9B). Instead, the empty fovea is anteriorly bordered by a thick and blunt process; process **Sp** short and slender and apically truncate (Figure 9B).

• Coxites I reduced, paracoxites I deeply modified, appearing as slender processes, distally slightly broadened, apically bearing a protruding mesal process and a highly reduced lateral one (Figures 8B; 9B; 10B; 12C and D).

• Mesomerites II (Figures 8B; 9B; 10B and 12A) much less sinuous and larger than mesomerites I of the ectopic gonopod on ring 8, almost rectilinear, distally bearing a large process (**mdp)** apically globular on the left mesomerite and articulated on the right (similar to mdp of mesomerites II of the ectopic gonopod on ring 8).

• A ‘rod-like’ mesal process (**rp**), broad and apically blunt, rising from the same basis as the mesomerite and almost reaching it in length, only on the left gonopod (Figures 9B and 11B).

• An additional small globular process (**vp**) with a tiny anterior projection, sitting at the basis of the mesal extension of the coxite, only on the left gonopod (Figure 9B).

• Solenomerites II larger than solenomerites I, the right one showing the three typical processes (**msp**, **Sp**, **La)** which look more or less normal (Figure 8B) while on the left solenomerite, the mesal slender process is missing or not fully individualized (Figure 9B), similar to the left solenomerite I of the ectopic gonopod on ring 8. However, here the antero-mesal process is more developed, longer, distally broader, apically strongly narrowed into a slender curved process (**msp**?). On the other hand, **msp** and **La** look more or less unmodified although **msp** is distally broken. Foveae empty.

• Coxites and paracoxites II (Figure 12) reduced, with seemingly broken apical lateral process.

**Figure 13:**
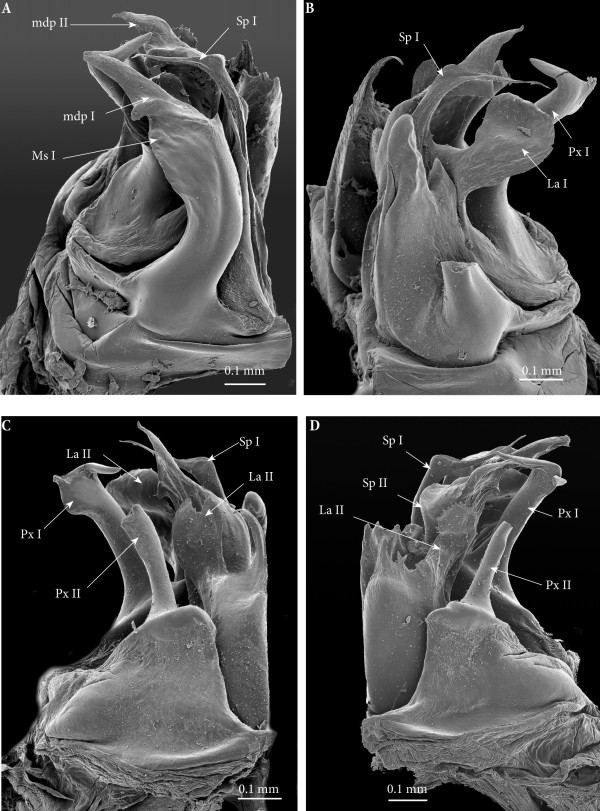
**Ectopic gonopod on ring 16, SEM. (A)** Left gonopod, antero-apical view. **(B)** Right gonopod, antero-apical view. **(C)** Left gonopod, posterior view. **(D)** Left gonopod, posterior view.

**Figure 14:**
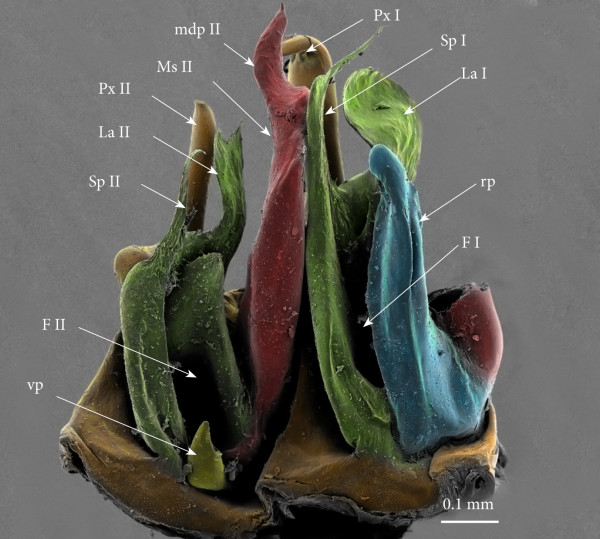
**Right ectopic gonopods on ring 16, mesal view, SEM, colours added.** Red, mesomerites; Green, solenomerites; Golden yellow, paracoxites; Bright yellow, vestigial process; Blue, rod-like process.

*Ectopic gonopods on ring 16* (Figures 5B; 9C; 10C; 11B; 13 and 14)*.* This set encompasses two consecutive connected distorted posterior gonopods presenting normal anterior-posterior polarity, asymmetrical and consisting (from anterior to posterior) of

• Mesomerites I distorted but still large (significantly larger and less sinuous than mesomerites I on ring 15) bearing a protruding and blunt process (**mdp)**, right mesomerite broken (Figures 13B and 14).

• Solenomerites I: left solenomerite well developed (Figure 13B), bearing the usual processes (**msp**, **Sp**, **La**); right solenomerite modified: **msp** absent, replaced by a large rod-like process (**rp**), apically rounded (similar in shape to the process on second gonopod of the preceding set, and separated from main solenomerite by a lateral incision and a triangular process. **La** and **Sp** normal (Figure 14). Foveae empty.

• Coxites and paracoxites I (Figure 13C and D) similar in shape to those on the ectopic gonopod of ring 15 but more reduced.

• Mesomerites II more or less the same size as mesomerites I, bearing a shorter process (**mdp)** narrowed apically, pointing anteriad and ending in an acuminate claw-like tip (Figures 10C and 14).

• Solenomerites II malformed and reduced on both sides (Figures 9C; 13C, D and 14): Right solenomerite consisting of a reduced, apically notched lamella (**La**); process (**Sp)** almost half as long as normal (not exceeding the lamella and lacking the distal bulge and the distal-most part), apically tapering and curved. Process (**msp)** absent (or not fully formed?) replaced anteriorly by a broad process intimately connected with the rest of solenomerite, mesally a vestigial process (**vp**) lays at the basis of the process, in front of the fovea. Left solenomerite very rudimentary, showing a small lateral lamella. Both **Sp** and **msp** are completely missing and replaced by a low, marginally serrated process connecting the lateral lamella (**La**) to the mesal margin of mesomerite. Foveae empty.

• Paracoxites II: slenderer and reduced, both apically broken at mid-length (Figures 10C; 13C and D).

## Discussion

Descriptive (non experimental) teratology has still much to offer to developmental biology (cf. [24,25]). There is a problem, however, in mining the literature for this kind of evidence, because the most interesting examples, those for which we could nowadays formulate tentative explanatory hypotheses in terms of gene (mis)expression, are often concealed under poorly informative terminology and are seldom described in satisfactory detail.

The specimen described in this paper is by far the most conspicuous and complex example of a male millipede with ectopic extra gonopods.

To offer an interpretation of the origin of the segmental distribution of these extra gonopods, we must first discuss, how to count serial features in a millipede, or, what to count? In this specimen, the mismatch between dorsal and ventral serial structures is even larger than usual in millipedes, as witnessed by the single ring bearing four pairs of walking legs. Under these circumstances, we suggest that it is sensible to integrate the information derived from both dorsal and ventral segmental patterns, to ‘reconstruct’ the whole sequence of segmentation steps.

Janssen et al.’s [1,26,27] investigations on the patterns of expression of segmentation genes in *Glomeris marginata* have shown that segmentation in millipedes is obtained independently (to some extent) in the dorsal vs. ventral halves of the trunk and only put in register after the segmental units, both dorsal and ventral ones, have been established.

Since the dorsal units are subject to fusion in pairs (local anomalies excepted), let’s identify positions by counting in terms of pairs of trunk appendages.

In our specimen there are two sets of gonopods, each of them involving four consecutive pairs of appendages: 8 to 11 for the anterior set, 24 to 27 for the posterior set. Thus, the posterior set is displaced by exactly 16 units, in respect to the anterior set, along the AP body axis. In our opinion, this suggest that a body section including 16 leg pairs could be a module deriving from 4 cycles of regular binary splitting of an embryonic ‘primary segment’.

**Figure 15:**
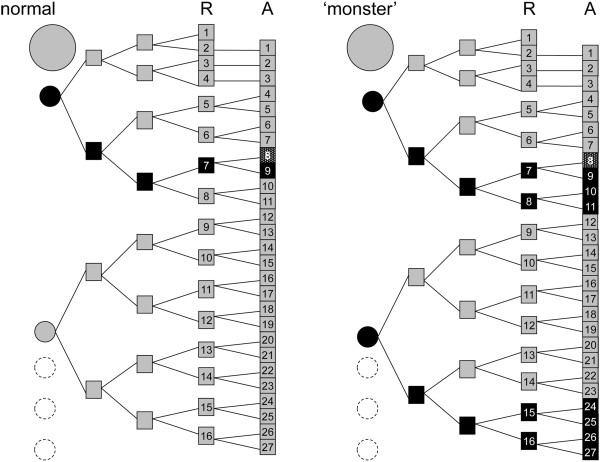
**Segmental composition of the anterior trunk in julidan millipedes.** Segmental composition of the anterior part of the body in the homeotic specimen of *Ommatoiulus moreleti* described in this paper, compared to a normal male, as suggested by the multiplicative segmentation model [15-17,28]. The first trunk rings are the legless collum and 3 rings with one leg pair each, followed by typical diplosegments. In the homeotic specimen, only one ring (13 + 14) corresponds to leg pairs 20–23. In each panel, the left vertical sequence of circles represents the embryonic head (big circle) followed by a few primary segments (smaller circles), the number of which is left indeterminate (fading empty circles toward the posterior). Each primary segment is subsequently split, through a sequence of binary AP divisions, into a growing number of secondary (definitive) segments. The intermediate stages from primary to secondary segments as well as the secondary segments (rings, R) and the associated pairs of appendages (A) are represented as squares. Appendage pairs are numbered.

A tentative interpretation for the segmental composition of the anterior part of the trunk of the homeotic specimen is provided in Figure 15.

The hypothesized segmentation pattern is the same in both a normal specimen and in our homeotic specimen (Figure 15). The difference between them would simply consist in the size of the embryonic trunk region endowed with a positional marker whose presence will later determine the replacement of walking legs by (posterior) gonopods.

We suggest that

(a) this part of the trunk derives from the two anteriormost ‘primary segments’ (small circles in left column)

(b) The symmetrical pattern of splitting exhibited by the second set (eventually giving rise to 2 × 2 × 2 × 2 units) is the ‘normal’ pattern of secondary segmentation which is ‘truncated’ in the anterior part of the first set, i.e. what eventually develops as the collum plus the three rings with one leg pair each. This hypothesized reduction of an otherwise stereotyped pattern of segmentation at the anterior (and possibly also the posterior) end of the trunk would parallel the pattern of secondary annulation of body segments in leeches [29]. In these annelids, most of the body segments present an identical pattern of annulation (with, e.g., three rings per segment in *Glossiphonia* but five in *Hirudo*), but the number of annuli per segment progressively decreases towards the fore end, with e.g. 4, 3, 3, 2, 2, 1, 1 in *Hirudo*

(c) The positional marker for gonopods is already present in a primary segment (black circle) before this eventual splits into secondary segments, but is inherited only by a posterior subset of the secondary segments to which it gives rise

(d) In the homeotic specimen, the positional marker corresponding to the future gonopods was more abundant and/or more extensively distributed, so that (1) it was present both in the first (as in normal specimens) and in the second of the primary segments and (2) in the progeny of both of these the marker was inherited by a number of secondary segments higher than those (two, corresponding to leg pairs 8 and 9) normally found marked within the progeny of the first primary segment.

We do not know the actual nature of the signal responsible for the ‘non-systemic metamorphosis’ [21] of a leg pair into a gonopod, although at least one transcription factor (Abdominal*-*B) can be hypothesized to be involved [21,29]. Indeed, the localization of external genital structures has been repeatedly shown to be controlled by *Hox* genes of the posterior (*Abdominal-B*) class, not only in arthropods such as the spider *Cupiennius salei*[30], but also mice [31] and *Caenorhabditis elegans*[32].

Still more, we do not know the ‘molecular code’ responsible for the difference between producing an anterior vs. a posterior gonopod. However, even in this respect our homeotic specimen is probably informative.

It is quite possible that multiple signalling, rather than the local expression of just one transcription factor, is responsible for the localization of millipede gonopods. However, the final balance of the local expression of the relevant genes will somehow depend on a strictly localized expression (or de-repression) of an instructive gene, or on the strictly localized repression of a more broadly expressed gene.

A different question is the specification of anterior vs. posterior gonopods, with the associated question of why all ectopic gonopods in the specimen described in this paper are (more or less complete) posterior gonopods, up to four consecutively.

It is likely that a molecular marker eventually releasing the non-systemic metamorphosis of walking legs into gonopods extends to the whole ring 7 of normal (male) julidan millipedes, where both anterior and posterior gonopods will be eventually formed. In our homeotic specimen, this marker is also found in other rings (their position relative to ring 7 being tentatively explained by our multiplicative model of segmentation). In the absence of further control, the gonopod-forming mechanism would produce posterior gonopods, whereas an additional, very strictly localized marker is required to modulate the gonopod-forming process towards forming a pair of anterior gonopods. In our specimen, this further hypothesized marker remains localized to the position (appendage pair 8) where normal anterior gonopods are formed, and does not extend into segmental positions were extra gonopods (all of the posterior type) are formed. This suggests that the marker of anterior gonopods is first expressed at a time when the segmentation of the relevant part of the trunk has been completed.

Thus, coupled with the hypothesis of an early determination of the sites of future non-systemic metamorphosis of walking legs into gonopods, the multiple duplication model of segmentation provides a detailed explanation for the segmental distribution of the extra gonopods in the homeotic specimen described in this paper. Alternative models, based on the sequential antero-posterior determination of individual segments or, at most, sets of two segments, (although a bisegmental pattern of expression of segmentation genes has not been discovered yet in millipedes), cannot explain the multiple occurrence, in the distribution of the extra gonopods, of spatial patterns based on different powers of 2: the set of 2 extra gonopods corresponding to legs 10 and 11; the sets of 4 gonopods each corresponding to legs 8–11 and 24–27, and the 16 segmental units (counted as leg pairs) separating the first (8 resp. 11) from the last (resp. 24 and 27) pair of appendages developed as gonopods in the described specimen.

Previous to our case, the literature dealing with teratological millipedes (largely summarized in [33]) included only three cases of homeotic males with ectopic gonopods.

In two instances, extra modified appendages were immediately contiguous with the (normal) gonopods, a circumstance probably caused by a localization of a positional marker (transcription factor or combination of transcription factors) less circumscribed than in normal specimens. Specifically, a teratological specimen of the polydesmidan species *Pseudoeurydesmus baguassuensis* Schubart, 1944 was found to possess the left leg 7 replaced by an extra gonopod [33]. The specimen also exhibited normal gonopods as appendage pair 8, as characteristic of polydesmidans. Similarly, a teratological specimen of *Ommatoiulus moreleti* from Madeira [34] had appendage pairs 8 and 9 in form of normal gonopods, followed by an atrophied leg 10 and an additional malformed posterior gonopod at the level of leg 11. As suggested by a reviewer of an earlier version of our paper, the asymmetry observed in both instances may well have been caused by a left/right phase shift in a posterior clock mechanism, but the two ‘monsters,’ as far as we can guess from the limited descriptions available in the literature, did not show anomalies in segment number or shape, but only in the kinds of appendages specified in the proximity of the normal gonopods. These two examples are arguably irrelevant for a discussion of segmentation mechanisms.

**Figure 16:**
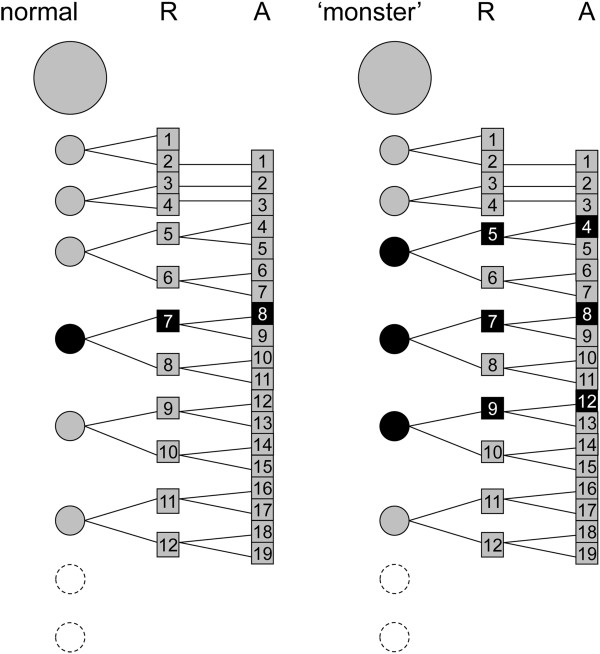
**Segmental composition of the anterior trunk in polydesmidan millipedes.** Segmental composition of the anterior part of the body in the homeotic specimen of *Nannaria conservata* described in [35], compared to a normal male, as suggested by the multiplicative segmentation model [15-17]. As in julidans (Figure 15), the first trunk rings are the legless collum and 3 rings with one leg pair each, followed by typical diplosegments. The number of secondary segments deriving from each primary segment is assumed to be lower (four rather than 16) in respect to the julidans, in agreement with the smaller total number of body rings in adult polydesmidans, compared to adult julidans.

Completely different is the case of a male of the polydesmidan *Nannaria conservata* Chamberlin, 1940 [35], with two additional pairs of gonopods in the place of legs 4 and 12, respectively, i.e. exactly 4 pairs in front and 4 pairs behind the normal pair of gonopods corresponding to appendage pair 8. As in *Nannaria*, and polydesmidans generally, the total number of the animal’s body rings is much lower than in julidans, it seems sensible to suggest that in the former lineage the hypothesized degree of segment multiplication is lower than in the latter. On this base we formulate a tentative interpretation of the segmentation process in normal polydesmidans and in the *Nannaria* specimen with two extra pairs of gonopods (Figure 16).

Alternatively to the suggested interpretation in terms of a putative multiplicative model of segmentation, we might try to fit the segmental distribution of normal and ectopic gonopods in our specimen and in the just discussed teratological specimen of *N. conservata*[35] with reference to the batches of body rings and/or trunk appendages progressively added with the first moults during the animal’s post-embryonic life. Specific developmental tables are not available for these two species, but we can confidently refer to the corresponding tables for other julids and polydesmids, as proxies for *O. moreleti* and *N. conservata* respectively [22]. In neither case can we find two consecutive stadia in which the relative positions occupied by gonopods in the two ‘monsters’ correspond to morphologically equivalent appendage pairs, e.g. the ultimate, or the penultimate one. This rules out the possibility to interpret the observed patterns in the same way as the previously mentioned discontinuous, multisegmental colour patterns observed in some millipede species [19].

Despite its circumstantial nature, the evidence provided by the specimen described in the present paper, as well as by the just discussed teratological specimen of *N. conservata*[34] suggests that the high or very high number of body segments achieved in the evolution of millipedes may involve segmentation mechanisms additional to those of which we have obtained understanding, mainly based on *Drosophila* and other model species with smaller, or much smaller, number of segments. The case of the two closely related *Scolopendropsis* species, one with 21 or 23 leg-bearing segments, the other with 39 or 43 [18], points in the same direction. If a multiplicative process of segmentation is actually involved in all these instances, it might well be that it rests on the iteration of a phylogenetically older, largely conserved mechanism elements of which we expect to be generally shared by arthropods.

## Material and methods

The homeotic specimen of *O. moreleti* was collected near Puerto de la Morcuera, Madrid, Spain, 20.9.1975, J. Tortajada Perote leg., and is preserved in 70% alcohol in the collections of the Museo Nacional de Ciencias Naturales, Madrid (MNCN 20.07/759). Measurements were made using a Leica Wild M10 microscope equipped with an ocular micrometer. Photos were taken with a Leica digital camera M205A mounted on a stereomicroscope Leica DFC 420, using Leica Application Suite software. For Scanning Electron Microscopy, the gonopods were cleaned with ultrasound, dehydrated in 96% ethanol and acetone, air-dried, mounted on adhesive electrical tape attached to aluminum stubs, coated with platinum/palladium and studied in a JEOL JSM-6335 F scanning electron microscope. Photos were processed in Leica Application Suite program and stacked in Zerene Stacker 1.04. All illustrations were edited using Adobe Lightroom 4.3, Adobe Photoshop CS.5, and assembled in plates with Indesign CS5.5.

## Abbreviations

Co: Coxite; F: Fovea; La: Lamella; Mdp: Meso-distal process of the mesomerite; Ms: Mesomerite; Msp: Meso-anterior process of the solenomerite; P: Promerite; Px: Paracoxite; Rp: Rod-like process on the solenomerite; Sp: Mesal slender process of the solenomerite housing the seminal groove; Spz: Spermatozoa; Vp: Vestigial process of the solenomerite.

## Competing interests

The authors declare that they have no competing interests.

## Authors’ contributions

NA carried out the morphological description and illustration. HE participated in interpretation of morphological observations and manuscript drafting. AM provided interpretation and discussion on the developmental aspects. All authors read and approved the final manuscript.
